# Estimation and Correlation of Serum Albumin and Serum Alkaline Phosphatase Levels Between Smokers and Non-smokers With Generalized Chronic Periodontitis

**DOI:** 10.7759/cureus.17474

**Published:** 2021-08-26

**Authors:** Himabindu Lalkota, Leela Subhashini C Alluri, Andre Paes Batista Da Silva, Sirisha Gummalla, Krishna Mohana Reddy Kondareddy

**Affiliations:** 1 Periodontology and Implantology, AME's Dental College and Hospital, Raichur, IND; 2 Periodontics, Case Western Reserve University School of Dental Medicine, Cleveland, USA; 3 Periodontics, Private Practice, Oklahoma, USA; 4 Periodontics, Private Practice, Hyderabad, IND; 5 Periodontology and Implantology, G. Pulla Reddy Dental College & Hospital, Kurnool, IND

**Keywords:** periodontal disease, alkaline phosphatase, serum albumin, smokers, non-smokers

## Abstract

Background

Periodontal disease is a chronic low-grade inflammatory disease triggered by periodontal microbial interaction present in the dysbiotic biofilm and the host's immune response further leads to the destruction of the supporting periodontal apparatus, which may, in turn, lead to tooth loss. Smoking is an environmental risk factor for periodontitis, and it enhances the secretion of various enzymes from host cells, which results in the initiation and progression of periodontal disease. The albumin concentration is related to nutrition and inflammation. Alkaline phosphatase (ALP), an enzyme found in various cells of the periodontium, is considered to cause the destruction of the periodontium. The study aimed to compare the serum albumin and serum ALP levels in smokers and non-smokers with generalized chronic periodontitis.

Materials and methods

The cross-sectional study included a total of 60 subjects. Subjects were divided into two groups, which included non-smokers with generalized chronic periodontitis (NS+P) and smokers with generalized chronic periodontitis (S+P). Clinical parameters analyzed were plaque index, gingival index, probing pocket depth, and clinical attachment level. The serum ALP and albumin levels were analyzed using a fully automated analyzer.

Results

The serum ALP levels were higher in the S+P group compared to the NS+P group. Conversely, the serum albumin levels were lower in the S+P group compared to the NS+P group.

Conclusion

There was a significant correlation of increased serum ALP levels and decreased serum albumin levels in the S+P group compared to the NS+P group.

## Introduction

Periodontitis is a chronic low-grade inflammatory disease affecting the teeth-supporting structures and ultimately leading to tooth loss [1]. Periodontal disease severity and progression are determined by numerous factors, including genetic, epigenetic, environmental factors (smoking, stress, and diet), aging, systemic diseases including diabetes, and immunological factors [2]. Smoking is the established risk factor for periodontal disease, with increased odds of disease progression in voluntary and involuntary smokers [3]. Several researchers have found an association between smoking habits and risk for the development of periodontitis [3-6]. Periodontal disease diagnosis is mainly based on assessing clinical parameters like probing pocket depth (PPD), bleeding on probing, clinical attachment level (CAL), and radiographic bone levels [7,8]. These clinical parameters are currently the only available indicators for current disease status and severity. However, they provide insights into past periodontium destruction and do not provide any information regarding the current status and future risk of attachment loss [7,8]. Furthermore, clinical measurements are subjective and have low specificity and sensitivity with a standard error of judgment of around + 1 mm. Thus, there is a constant search for biomarkers that will objectively and quantitatively assess periodontal disease extent and severity [7,8]. Biomarkers are reliable and reproducible measures for discrimination between disease and health to determine disease activity. However, it is practically impossible that a standalone biomarker will fulfill all the criteria for a diagnostic tool [7,9]. Previous periodontal research has evaluated many such biomarkers, which positively associate with periodontitis. Alkaline phosphatase (ALP) was one of the first enzymes identified as an indicator of periodontitis [10]. They are plasma membrane-bound glycoproteins produced by different cells of the periodontium. They have been released from polymorphonuclear cells, periodontal ligament fibroblasts, and osteoblast in periodontal regeneration during the inflammatory process [11-13]. Evidence from previous research suggests that ALP activity increases during gingivitis and periodontitis locally in gingival crevicular fluid (GCF) and saliva [14,15]. However, studies evaluating the increased serum ALP during periodontitis are limited. Evidence suggests that there is a link between periodontitis and systemic inflammation. Thus, research is focused on evaluating systemic biomarkers of inflammation in periodontitis.

The serum albumin is primarily synthesized in the liver, and its concentration is an empirical biomarker of general systemic health status as it determines the severity of the ongoing diseases [16]. On the other hand, relationship between periodontitis and general health status has been established [17]. Yoshihara et al. were the first to establish an association between oral health and serum albumin [18]. Ogawa et al. in 2006 found an association between periodontitis and the elderly [16,19]. However, studies evaluating the association between serum albumin and smoker with (or) without periodontitis are scarce. Consequently, previous studies conducted have evaluated the association between periodontitis in elderly patients only. So, it is crucial to find its association in middle-aged adults too.

Thus, it was hypothesized that smoking has effects on periodontal inflammation, and, therefore, serum ALP levels could indicate the current periodontal disease activity and serum albumin levels the general health status in periodontitis patients. Thus, the study was designed to estimate and correlate the serum ALP and serum albumin levels in smokers and non-smokers with generalized chronic periodontitis.

## Materials and methods

The present study was conducted on 60 male patients aged 30-65 years in the Department of Periodontology and Implantology, G. Pulla Reddy Dental College and Hospital, Kurnool. The Institutional Ethical Committee approved the study. Furthermore, the informed consent was signed by the subjects. Of these 60 patients, 30 subjects were smokers associated with generalized chronic periodontitis (S+P) and 30 subjects were non-smokers associated with generalized chronic periodontitis (NS+P). The exclusion criteria include female patients, patients with a history of systemic diseases (uncontrolled or poorly controlled diabetes, unstable or life-threatening conditions), and patients under anti-inflammatory/antibiotic therapy for the previous six months because the pathophysiology for periodontitis was altered in these diseases.

**Figure 1:**
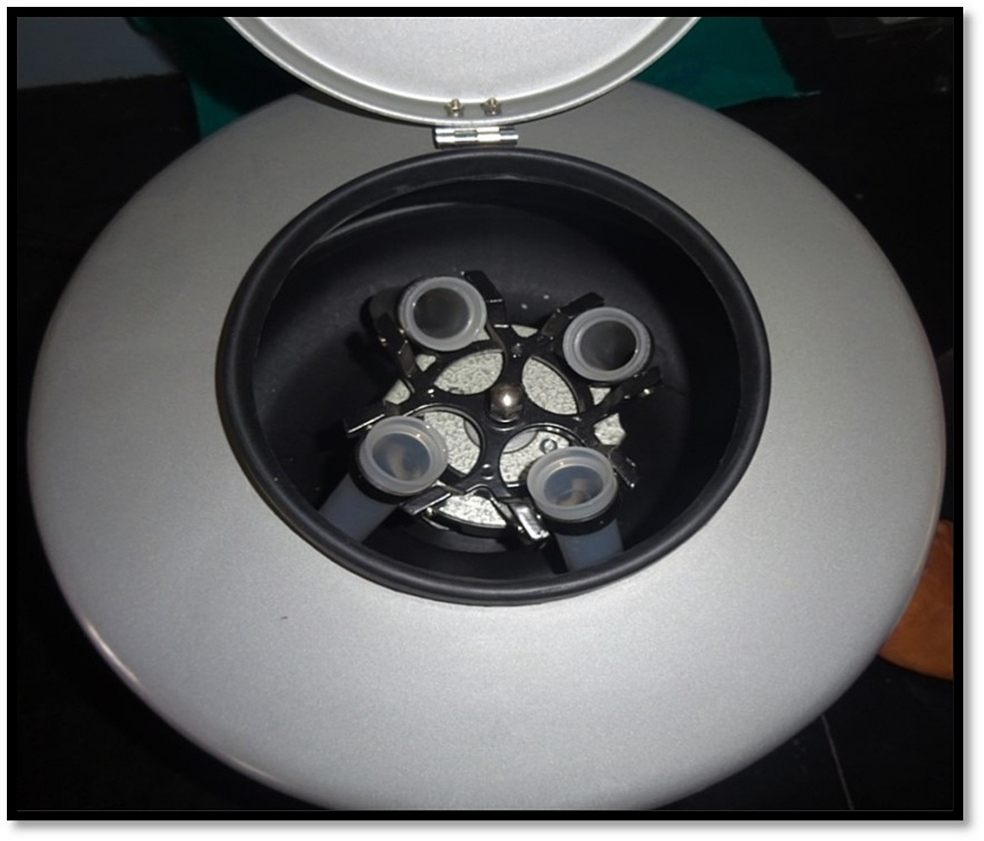
Centrifuge Machine

The inclusion criteria include subjects aged 30-65 years and patients with 3-4 mm of CAL. Patients with a history of smoking are included in the (S+P) group, and patients who have never smoked in their lifetime are included in the (NS+P) group. For clinical examination, a minimum of 18 teeth in each subject were examined. PPD, gingival index (GI), plaque index (PI), and CAL were recorded at four sites of each tooth of all teeth using William’s periodontal probe. One examiner recorded all the clinical data. The supragingival plaque was scored using the PI [20]. Gingival inflammation was scored using the GI [21]. Two milliliters of venous blood was drawn by venipuncture. Blood was collected in the test tube, which was allowed to stand for 30 minutes at room temperature and centrifuged at 3000 rpm for 5 minutes, and separate serum from it (Figure 1).

**Figure 2:**
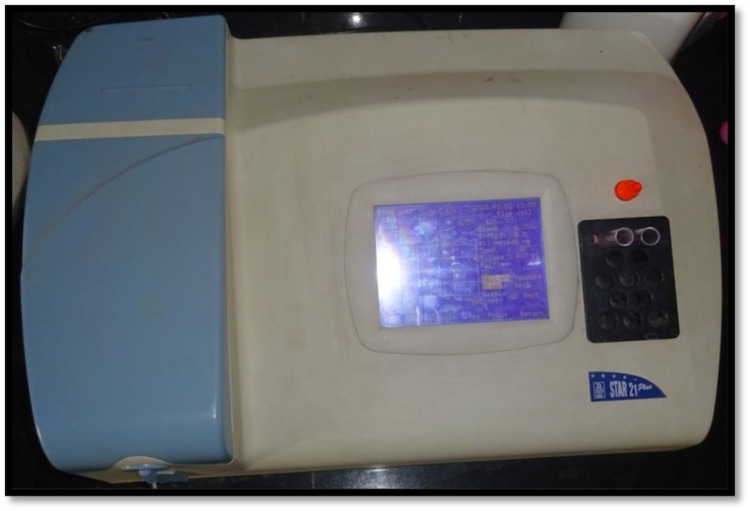
Autoanalyzer (Star 21 Plus)

**Figure 3:**
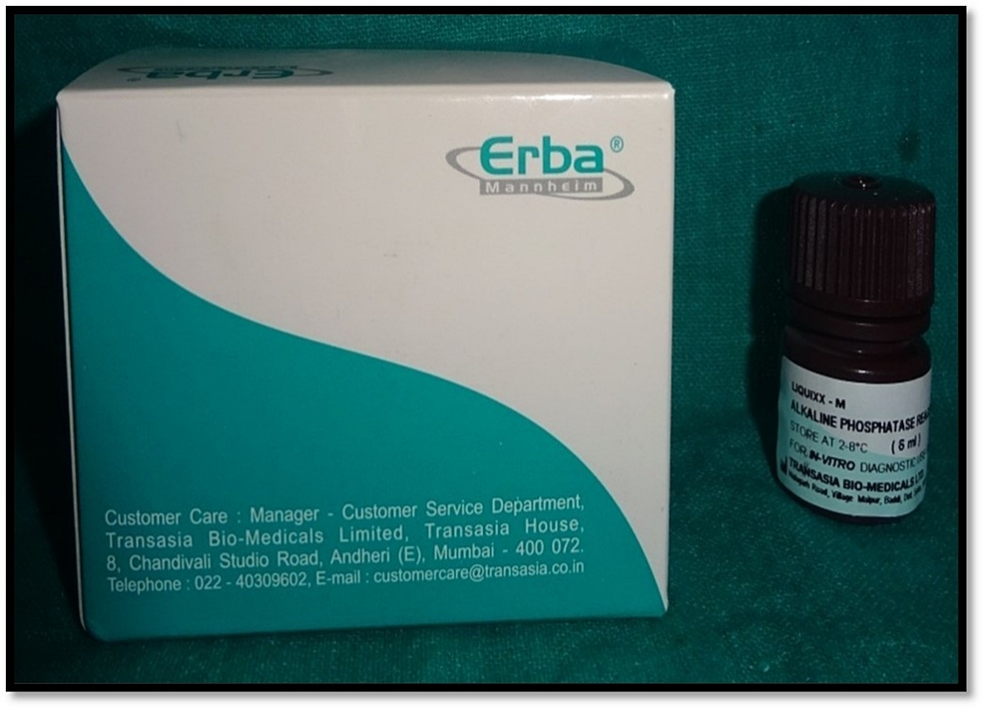
Alkaline Phosphatase Reagent Kit

**Figure 4:**
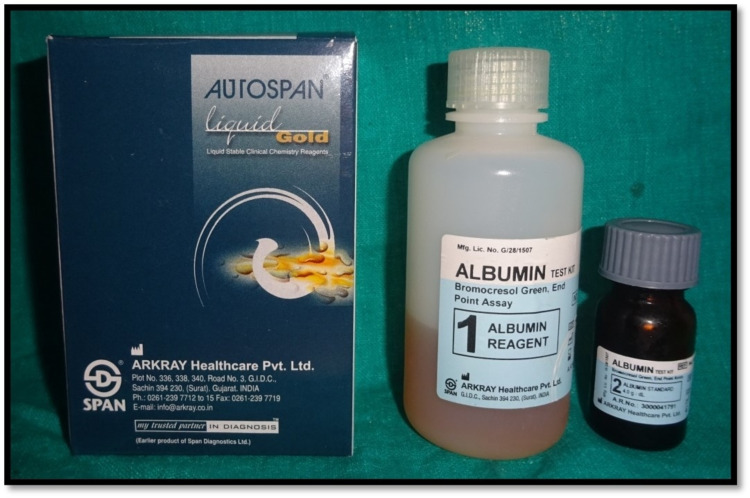
Albumin Reagent Kit

**Figure 5:**
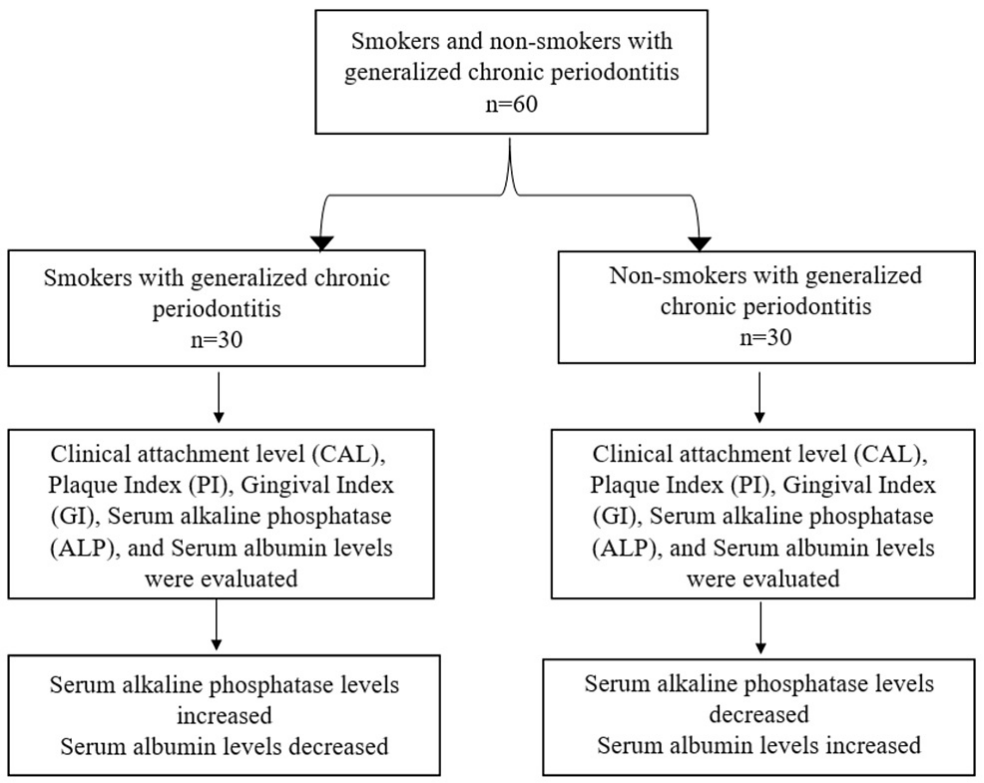
Schematic Representation of the Study Design

Serum was analyzed using Star 21 Plus fully automated auto-analyzer (Figure 2) to estimate ALP and albumin levels. The Erba serum ALP kit (Figure 3) estimated the biochemical value of serum ALP, and serum albumin level was measured by the bromocresol green albumin method (Figure 4). The schematic representation of the study design is illustrated in Figure 5.

 

 

 

Statistical analysis

The data were calculated for mean and standard deviation. The statistical significance of the difference between groups has been tested with Student's paired t-test.

## Results

The PI and GI, which are essential indicators for periodontal health and generalized chronic periodontitis, showed grossly different values between the two groups. The mean value and standard deviation of the PI score in (S+P) and (NS+P) were 1.542±0.510 and 0.937±0.134, respectively. The mean value and standard deviation of GI scores in (S+P) and (NS+P) were 1.527±0.226 and 2.103±0.313, respectively. In comparison, the differences between the PI and GI values in (S+P) and (NS+P) were found to be statistically significant (p < 0.0001) (Table 1).

**Table 1:** Comparison of Mean Plaque Index, Gingival Index, and Clinical Attachment Level Between S+P and NS+P S+P, smokers with generalized chronic periodontitis; NS+P, non-smokers with generalized chronic periodontitis. *p < 0.05 statistically significant.

Plaque Index			Gingival Index		Clinical Attachment Level	
	S+P	NS+P	S+P	NS+P	S+P	NS+P
Mean	1.542	0.937	1.527667 (42%)	2.103667 (58%)	3.496667	2.523333
SD	0.510222	0.134091	0.226041	0.313	0.268756	0.248281
t-Value	6.281360928		8.171426222		14.57051199	
p-Value	0.00001*		0.00001*		0.00001*	

The mean value and standard deviation of CAL scores in (S+P) and (NS+P) were 3.496±0.268 and 2.523±0.248 mm, respectively. In comparison, CAL scores between (S+P) and (NS+P) were found to be statistically significant (p < 0.0001) (Table 1). The mean value and standard deviation of serum albumin in (S+P) and (NS+P) were 2.925±0.563 and 3.253±0.619 g/dL, respectively. In comparison, the serum albumin between (S+P) and (NS+P) was found to be statistically significant (p < 0.0001) (Table 2).

**Table 2:** Comparison of Mean Serum Albumin and Serum ALP Levels Between S+P and NS+P ALP, alkaline phosphatase; S+P, smokers with generalized chronic periodontitis; NS+P, non-smokers with generalized chronic periodontitis. *p < 0.05 statistically significant.

Serum Albumin			Serum ALP	
	S+P	NS+P	S+P	NS+P
Mean	2.925333	3.253333	94.46667	58.36667
SD	0.563785	0.619507	21.98233	15.66785
t-Value	2.144750274		7.324737654	
p-Value	0.03617372*		0.00001*	

The mean value and standard deviation of serum ALP in (S+P) and (NS+P) were 94.466±21.982 and 58.366±15.667 µ/mL, respectively. In comparison, the serum ALP between (S+P) and (NS+P) was found to be statistically significant (p < 0.0001) (Table 2).

## Discussion

Periodontal diseases are chronic inflammatory conditions with prevalence rates ranging from 5.5% to 85.1% adult population [22]. Subsequent activation of the patient’s host response leads to the release of various metabolic byproducts at the tooth and periodontal pocket interfaces, such as destructive cellular enzymes, cytokines, chemokines, and other pro-inflammatory mediators of tissue destruction [23]. Multiple markers in GCF, saliva, and serum have been evaluated as a diagnostic tool for periodontal diseases with high specificity and sensitivity. However, the presence of a single standalone biomarker in periodontitis is unlikely due to its complex mechanism. Thus, there is a constant search for different combinations of biomarkers in periodontal research [23]. The results of the present study concerning the smoking group contradict previous studies done by Patil et al. [24], and Kibayashi et al. [25] found that ALP activity decreased in the smoking group compared to non-smoking groups' periodontitis. Kibayashi et al. attributed a decrease in ALP activity to impaired neutrophil function in smokers leading to impairment of an inflammatory response in the development of periodontitis due to current smoke exposure [25]. The discrepancy in findings of the present study with that of previous studies could be because of variable study design, the inclusion of study groups, data analysis, and samples analyzed. Previous studies performed had used either saliva or GCF as samples representing local pathologic changes compared to the serum, a marker of systemic changes. Thus, it can be speculated that the increase in serum ALP in S+P could be because of the systemic effects of smoking on ALP levels. This was substantiated in Wannamethee and Shaper's study, which found that cigarette smoking was significantly associated with increased serum ALP levels irrespective of alcohol usage [26]. Similar results were obtained by Jang et al., who found increased ALP in smokers and attributed its increase to inflammation and increased bone turnover [27]. However, these mechanisms are speculative, and further studies need to be conducted for conclusive evidence.

The present study demonstrated a positive correlation between serum ALP and the clinical parameters (PI, GI, PPD, and CAL). The strongest correlation was found in smokers with periodontitis, followed by non-smokers with periodontitis. Furthermore, the mean serum ALP concentration was significantly higher in the S+P group than the NS+P group. The present study's results agree with previous studies done by Malhotra et al., who found a positive correlation between ALP activity and the clinical parameters [28]. Concerning serum albumin levels, S+P group revealed the lowest albumin levels compared to other groups. The results of the present study showed an inverse independent relation between periodontitis and albumin levels. The results of the present study agree with Kolte et al., who found lower albumin levels in patients with chronic periodontitis [29]. However, the results of the present study need to be cautiously compared with Kolte et al.'s because of the different age groups included and the smoking status. The age group included in our study was 26-60 years compared to 40-70 years in the previously mentioned studies. This is particularly important because albumin levels are gradually lowered in older age groups because of impaired dentition status and a lean lifestyle and the possibility of compromised systemic health status. Similar results were reported by Ogawa et al., where albumin concentration was lowered in patients above 70 years of age who were diagnosed with periodontitis [19]. However, the exact mechanism behind serum albumin-periodontitis link is unknown. It is difficult to speculate whether the serum albumin concentration is genuinely affected by the inflammatory component of periodontitis or the individuals' general health or nutritional status. However, the effect of nutritional status or general health was somewhat excluded from our study as the participants were a systemically healthy, middle-aged group with mean teeth present per subject being 25. Thus, it can be hypothesized that lower serum albumin concentration might have possibly been affected by the inflammatory component of periodontitis.

The present study demonstrated lower serum albumin levels in S+P as compared to NS+P. Although this difference was not significant, the range was lower in the smokers' group. This suggests that smoking interferes with serum albumin concentration. In a study done by Shaper et al., it was concluded that cigarette smoking has an inverse relationship with serum albumin concentration [30]. To the best of our knowledge, this is the first study evaluating the effect of smoking on serum albumin concentration in patients with periodontitis. Thus, further longitudinal studies need to be conducted to provide sufficient evidence supporting the results of the present study. The results of the present study need to be cautiously interpreted considering several limitations of the study. The present study was conducted in Indian population only; thus, future studies including participants from different races and ethnic groups need to be conducted. Another factor that might have affected the results is gender variability in the present study, as the number of males was considerably more significant in the smoking group. Furthermore, the study has been performed before non-surgical periodontal therapy (NSPT), although after NSPT, values need to be evaluated.

## Conclusions

The present study evaluated and correlated the serum albumin and serum ALP levels between smokers and non-smokers with generalized chronic periodontitis. There are many studies on detecting albumin and ALP levels in GCF and saliva, whereas there are few studies that detect albumin and ALP levels in serum. Serum albumin and serum ALP levels can be detected in patients with generalized chronic periodontitis and used as biomarkers. Furthermore, there was a significant correlation of increased serum ALP levels and decreased serum albumin levels in smokers with generalized chronic periodontitis compared to non-smokers.
